# Novel compound heterozygous mutations in the *OTOF* Gene identified by whole-exome sequencing in auditory neuropathy spectrum disorder

**DOI:** 10.1186/s12881-017-0400-0

**Published:** 2017-03-23

**Authors:** Fengzhu Tang, Dengke Ma, Yulan Wang, Yuecai Qiu, Fei Liu, Qingqing Wang, Qiutian Lu, Min Shi, Liang Xu, Min Liu, Jianping Liang

**Affiliations:** 1 Department of Otolaryngology, The People’s Hospital of Guangxi Zhuang Autonomous Region, 6 Taoyuan Road, Nanning, 530021 China; 2 CapitalBio Technology Co., Ltd., Building C, Block 88 Kechuang 6th Street, Yizhuang Biomedical Park, Beijing Economic- Technological Development Area, Beijing, 101111 China; 3 Research Center of Medical Sciences, The People’s Hospital of Guangxi Zhuang Autonomous Region, 6 Taoyuan Road, Nanning, 530021 China

**Keywords:** Auditory neuropathy spectrum disorder, Heterozygous mutations, *OTOF* gene, Whole-exome sequencing

## Abstract

**Background:**

Many hearing-loss diseases are demonstrated to have Mendelian inheritance caused by mutations in single gene. However, many deaf individuals have diseases that remain genetically unexplained. Auditory neuropathy is a sensorineural deafness in which sounds are able to be transferred into the inner ear normally but the transmission of the signals from inner ear to auditory nerve and brain is injured, also known as auditory neuropathy spectrum disorder (ANSD). The pathogenic mutations of the genes responsible for the Chinese ANSD population remain poorly understood.

**Methods:**

A total of 127 patients with non-syndromic hearing loss (NSHL) were enrolled in Guangxi Zhuang Autonomous Region. A hereditary deafness gene mutation screening was performed to identify the mutation sites in four deafness-related genes (*GJB2*, *GJB3*, 12S rRNA, and *SLC26A4*). In addition, whole-exome sequencing (WES) was applied to explore unappreciated mutation sites in the cases with the singularity of its phenotype.

**Results:**

Well-characterized mutations were found in only 8.7% (11/127) of the patients. Interestingly, two mutations in the *OTOF* gene were identified in two affected siblings with ANSD from a Chinese family, including one nonsense mutation c.1273C > T (p.R425X) and one missense mutation c.4994 T > C (p.L1665P). Furthermore, we employed Sanger sequencing to confirm the mutations in each subject. Two compound heterozygous mutations in the *OTOF* gene were observed in the two affected siblings, whereas the two parents and unaffected sister were heterozygous carriers of c.1273C > T (father and sister) and c.4994 T > C (mother). The nonsense mutation p.R425X, contributes to a premature stop codon, may result in a truncated polypeptide, which strongly suggests its pathogenicity for ANSD. The missense mutation p.L1665P results in a single amino acid substitution in a highly conserved region.

**Conclusions:**

Two mutations in the *OTOF* gene in the Chinese deaf population were recognized for the first time. These findings not only extend the *OTOF* gene mutation spectrum for ANSD but also indicate that whole-exome sequencing is an effective approach to clarify the genetic characteristics in non-syndromic ANSD patients*.*

**Electronic supplementary material:**

The online version of this article (doi:10.1186/s12881-017-0400-0) contains supplementary material, which is available to authorized users.

## Background

Deafness is one of the most common clinical otological diseases, and most cases are caused by genetic factors [1, 2]. Hereditary hearing loss has high genetic heterogeneity, non-syndromic hearing loss (NSHL) has only the symptom of deafness and accounts for 70% of all hereditary deafness, while syndromic deafness accounts for 30% of all cases [1, 2]. The majority of NSHL has been confirmed to have Mendelian inheritance from single gene mutations, and these cases mainly include autosomal recessive or autosomal dominant transmission and X-linked or mitochondrial inheritance [3]. More than 90 genes related to NSHL have been identified. Earlier studies have shown mutations in several genes that are closely related to NSHL in China, such as mtDNA 12S-rRNA, *SLC26A4*, *GJB2*, *GJB3*, and *GJB6* [4], while the causes of disease in other deaf individuals remain unknown.

Whole-exome sequencing (WES) is a novel technique that has offered a breakthrough for studying rare Mendelian diseases that allows for sequencing of all expressed genes in the genome, which is substantial considering the protein-coding regions covers approximately 85% of human disease-causing mutations [5]. WES is a novel technique of genome analysis that has the advantages of being simple, economical and accurate, and it has become the most efficient way to identify genetic variants in all genes for an individual [6].

Auditory neuropathy spectrum disorder (ANSD) is considered as a type of autosomal recessive non-syndromic hearing loss in which the transmission of sound signals from the inner ear to the auditory nerve and brain is impaired. In the present study, we applied whole-exome sequencing of two affected siblings with ANSD and their healthy sisters from a Chinese family in Guangxi Zhuang Autonomous Region. For the first time, two compound heterozygous mutations, c.1273C > T (R425X) and c.4994 T > C (L1665P), in the *OTOF* gene were identified as disease-causing mutations in the Chinese deaf population.

## Methods

### Study design

A total of 127 patients who were diagnosed as NSHL were enrolled at The People’s Hospital of Guangxi Autonomous Region. Peripheral blood samples were collected for genomic DNA. Audiologic tests were performed as necessary. The audiologic tests include auditory brainstem response (ABR), auditory steady-state response (ASSR), distortion product otoacoustic emission (DPOAE) and cochlear microphonic (CM) tests.

### Hereditary deafness gene mutation screening and whole-exome sequencing

All the 127 cases enrolled in our study were subjected to a preliminary screening using a microarray method with a Hereditary Deafness Gene Mutation Detection Kit (CapitalBio Technology Co., Ltd. Beijing, China). The kit was designed to detect fifteen well-documented mutations in four deafness-associated genes, including *GJB2*: 35delG, 176_191del16, 235delC, 299_300delAT; *GJB3*: 538C > T; 12S rRNA: 1555A > G, 1494 T > C; and *SLC26A4*: IVS7-2A > G, 2168A > G, 1174A > T, 1226G > A, 1229C > T, 1975G > C, 2027 T > A, and IVS15 + 5G > A. Furthermore, The subsequent whole-exome sequencing was applied to the cases with singularity of its phenotype as following standard protocol by CapitalBio Technology Co., Ltd. Genomic DNA was extracted using a RelaxGene Blood DNA System (TIAGEN Biotech Co., Ltd. Beijing, China). Libraries were prepared using the Ion AmpliSeq™ Exome RDY Kit (Thermo Fisher Scientific Inc. Shanghai, China). We used the Ion OneTouch™ 2 System (Thermo Fisher) to prepare enriched, template-positive Ion PI™ Ion Sphere™ particles (ISPs) containing clonally amplified DNA. The template was sequenced on the Ion Proton™ System (Thermo Fisher) with the Ion PI™ Chip Kit v3 (Thermo Fisher). The average sequencing depth of 86.1× provided sufficient depth to identify variants for 90.9% of the targeted exome.

### Variant analysis

The coverage analysis plugin and variant caller plugin from Life Technologies (Thermo Fisher) were used to analyze the Ion Proton sequencing run. Variant discovery, genotype calling of multi-allelic substitutions and indels were performed on each individual sample using the Torrent Variant Caller (TVC, version 4.6.0.7) (Thermo Fisher). Statistics and graphs describing the level of sequence coverage produced for targeted genomic regions were provided by the Torrent Coverage Analysis (version 4.6.0.3). The variants were annotated by the Annotate variants 5.0 of Ion Reporter (Thermo Fisher).

### Direct Sanger sequencing

The potential causative variants in the family were confirmed by Sanger sequencing of amplified PCR products. Primer sequences for the pathogenic variant in the *OTOF* gene (NM_194248.2) were designed using Primer Premier 5.0 as follow: c.1273-F 5′- GGGAATCAATGAATCCTGTCT-3′ and c.1273-R 5′- TCCACGAGGTCCTTGTTTT-3′, c.4994-F 5′- GGTAGACAGGTGATGGCATAG-3′ and c.4994-R 5′- TGTCAAGGACCCAGTTCATC-3′.

## Results

### Clinical data and audiological assessments

Through the preliminary hereditary deafness gene mutation screening, previously well-characterized mutations were found in only 8.7% (11/127) of the patients with non-syndromic hearing loss (NSHL) enrolled in our study, which was described in our earlier work [7].

**Fig. 1:**
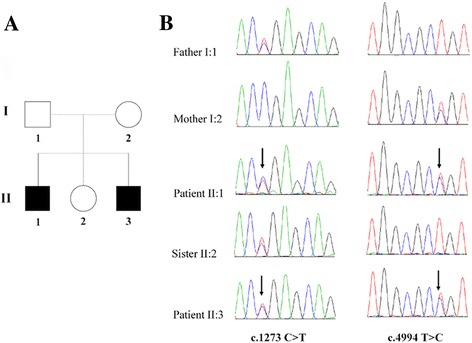
Pedigree and sequence analysis of *OTOF* mutations in the ANSD family. **a** In a Chinese ANSD family, compound heterozygous mutations c.1273 C > T and c.4994 T > C were observed in both affected siblings (II:1 and II:3); **b** One heterozygous mutation c.1273 C > T was observed in the sister (II:2). The c.1273 C > T heterozygous mutation is from the father (I:1), and the other heterozygous c.4994 T > C mutation is from the mother (I:2)

**Fig. 2:**
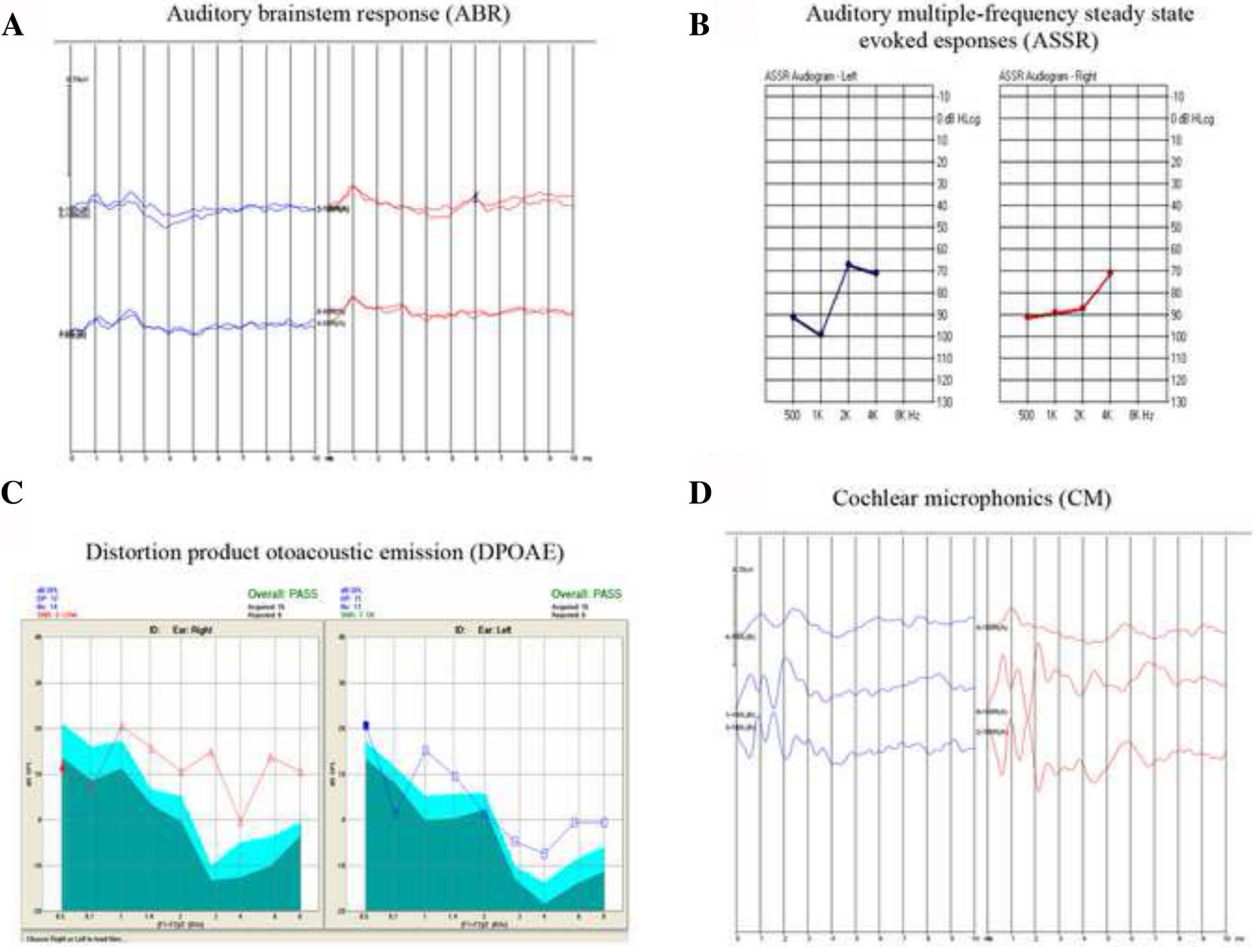
Audiologic tests of subject II:3. Subject II:3 was diagnosed with ANSD according to the audiological assessments; **a** ABR, auditory brainstem responses; **b** ASSR, auditory steady-state response; **c** DPOAE, distortion product otoacoustic emissions; and **d** CM, cochlear microphonics

In a family with NSHL collected in this study, the parents had normal hearing, while their two sons had abnormal hearing, and their daughter had normal hearing (Fig. 1a). Both hearing impaired subjects II:1 and II:3 presented with abnormal pronunciation and poor responses to sound. The general physical examinations of the two siblings were normal. Magnetic resonance imaging (MRI) showed that the structures of the inner ear in subjects II:1 and II:3 were normal (Additional file 1: Figure S1). The audiological assessments revealed that both two siblings had bilateral sensorineural deafness. The results of audiological assessments of the two affected subjects are summarized in Table 1 and the results for II:3 are shown in Fig. 2a, b, c, and d.

**Table 1:** Audiological assessments of the affected subjects

Subjects	Age	Sex	ASSR	DPOAE	ABR	CM
II:1	18	M	Bil profound	Present	Absent	Present
II:3	3	M	Bil profound	Present	Absent	Present

*ASSR* auditory steady-state response, *ABR* auditory brainstem responses, *Bil* bilateral, *DPOAE* distortion product otoacoustic emissions, *CM* cochlear microphonics, *F* female; and M, male

The affected subject II:1 underwent a right-side cochlear implant at the age of 18, and his pronunciation and response to the sound had improved since then. In addition, the affected subject II:3 had worn hearing aids in both ears since the hearing screening at the time of birth, but his ability to communicate was not improved. At 20 months of age, he underwent right-side cochlear implantation at The People’s Hospital of Guangxi Zhuang Autonomous Region. The surgery was successful, and no postoperative complications were observed. Then, he underwent speech rehabilitation training for more than 1 year. His speech was excellent, and he had a good response to sounds and therefore he has been enrolled in regular kindergarten. According to the audiological assessments and the history of the treatments and the outcomes, the two siblings were clinical diagnosed as ANSD.

### Whole-exome sequencing and variant analysis

Of all the patients evaluated in our study with non-syndromic hearing loss (NSHL), well-known mutations in four deafness-associated genes (*GJB2*, *GJB3*, 12S rRNA, and *SLC26A4*) were only detected in 8.7% (11/127) of the patients [7], suggesting that unappreciated genetic abnormalities may contribute to hearing loss diseases. We then introduced whole-exome sequencing to identify the novel mutations in the subjects of interest. A summary of the whole-exome sequencing data for the three samples of the family are shown in Table 2. There were 95,317,441 total reads, and there was an average read length of 179 bp. The total number of bases was 17.1 G, and 98% of the sequenced bases mapped to the human reference genome [8]**.**

**Table 2:** Whole-exome sequencing of the three siblings

Parameter	II:1	II:2	II:3
Total reads	29,611,231	29,893,511	33,929,246
Number of mapped reads	29,123,517	29,600,865	33,598,112
Percent reads on target	88.85%	89.52%	91.11%
Uniformity of base coverage	94.32%	95.08%	94.18%
Mean Read Length	176 bp	181 bp	181 bp
Mean read depth	79.68	82.84	95.77
Target base coverage at 20×	91.96%	93.78%	93.78%
Total number of variants	37918	37729	38169

The variant calling pipeline of Life Technologies was used to analyze the Ion Proton sequencing run. Ion Proton identified (37,918; 37,729; and 38,169) SNPs among three samples of the family (Table 2). The SNPs were annotated by the Annotate variants 5.0 of Ion Reporter (Life Technologies). Known variants with minor allele frequency (MAF) >1% were removed. The variants were filtered according to the inheritance model (autosomal recessive with both homozygous and compound heterozygous) and variant function categories, including missense, nonsense and frameshift mutations. The pathogenicity predictor softwares SIFT and Polyphen were used to predict the novel missense variant.

We then focused our attention to the variants that were associated with reported deafness genes. Two compound heterozygous mutations of the *OTOF* gene (R425X and L1665P) were identified in the deaf patients in the family (Fig. 1b). The novel missense variant reported in our study has not been found in dbSNP database (https://www.ncbi.nlm.nih.gov/projects/SNP/), 1000-Genomes Project (http://phase3browser.1000genomes.org/index.html) and NHLBI Exome Sequencing Project (http://evs.gs.washington.edu/EVS).

### Identification of pathogenic mutations

**Fig 3:**
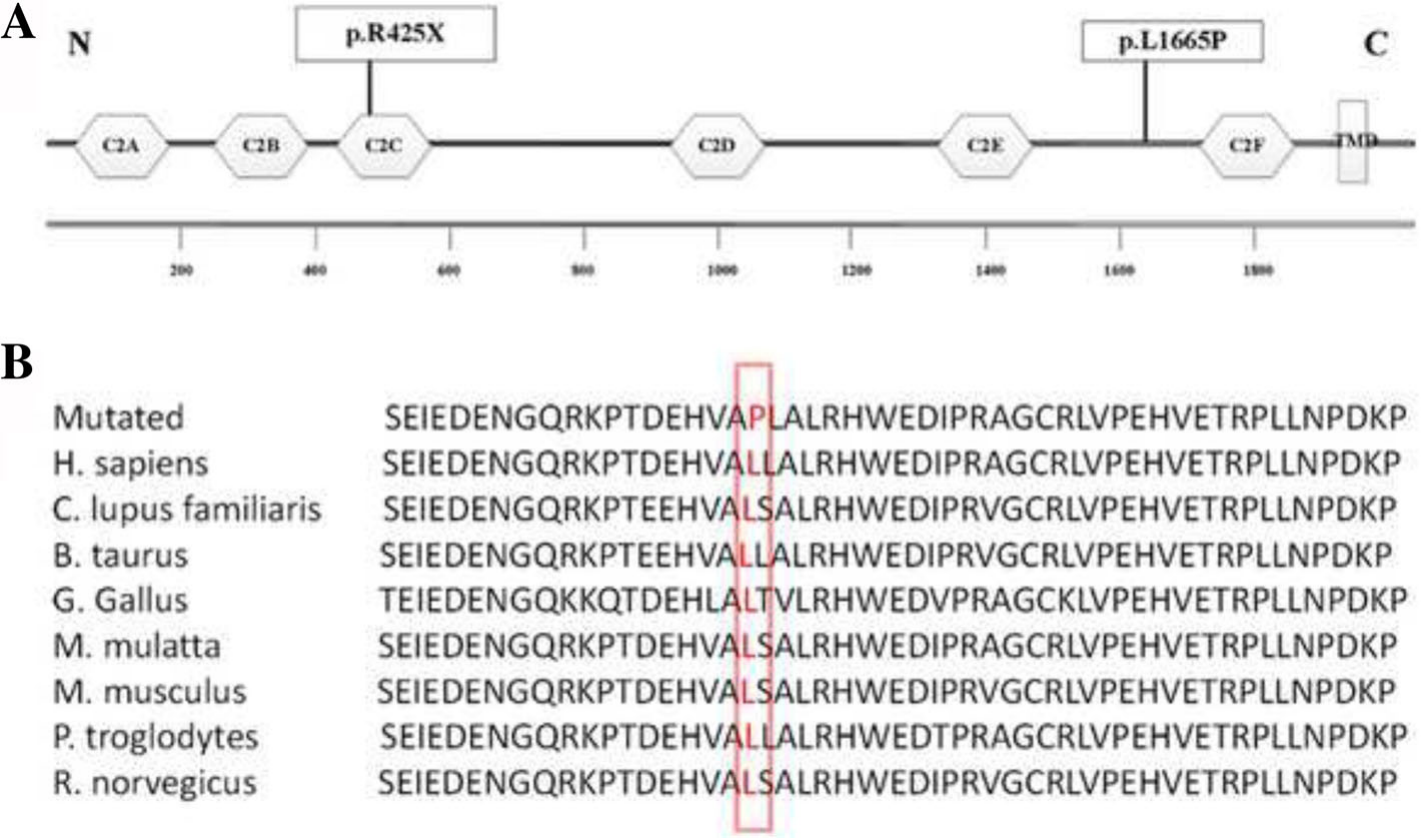
Schematic structure of otoferlin and conservation analysis. **a** The functional domains of otoferlin and location of the mutations identified in the study. **b** Conservation of the L1665P of otoferlin. TMD: the transmembrane domain

The c.1273C > T (p.R425X) mutation in the *OTOF* gene contributes to a premature stop codon may result in a truncated polypeptide that strongly suggests its contribution to the pathogenicity for ANSD (Fig. 3a). The amino acid L1665 is conserved across mammalian species, including humans, rats, dogs, mice and chimpanzees (Fig. 3b); c.4994 T > C (p.L1665P) was predicted to be harmful.

Two mutations of the *OTOF* gene (p.R425X and p.L1665P) were verified in the ANSD family by Sanger Sequencing. The results validated that both compound heterozygous mutations of the *OTOF* gene can be detected in the two affected siblings; however, their parents and sister were heterozygous carriers of c.1273C > T (father and sister) and c.4994 T > C (mother), showing a complete match of the genotype with the phenotype (Fig. 1a).

## Discussion

Although the majority of NSHL cases have confirmed Mendelian inheritance caused by single gene mutations, many deaf individuals do not have identified genetic contributions. WES provides a highly efficient strategy to identify the genetic variation that is responsible for Mendelian diseases. This study identified two disease-causing mutations in the *OTOF* gene in a Chinese family with ANSD by WES. The mutations in this gene were observed in the Chinese deaf population for the first time.

Auditory neuropathy is viewed as a variety of hearing loss in which the outer hair cells within the cochlea are usually functional, but the sound signals are unable to be accurately transmitted to the auditory nerve and brain also known as auditory neuropathy/auditory dys-synchrony (AN/AD) or auditory neuropathy spectrum disorder (ANSD). Audiology characteristics of ANSD contain severely abnormal or absent ABRs that begin with the VIII nerve component of wave I and preserve normal or near-normal otoacoustic emissions. ANSD can occur independently or be accompanied by other neurological symptoms, such as Charcot-Marie-Tooth disease [9]. The causes of ANSD are complex, which mainly include specific damage to the inner hair cells, synaptopathies and VIII nerve disorders. Genetic factors nearly account for 40% of all ANSD cases [10]. Four loci have been identified to account for non-syndromic ANSD; the mutations in the *OTOF* and *PJVK* genes cause autosomal recessive ANSD, the mutations in the *AUNA1* causes autosomal dominant ANSD, and the mutations in the *AUNX1* causes X-linked ANSD [10–15].

*OTOF* was first identified using a candidate gene approach and a positional cloning strategy in 1999. This 48-exon gene, encoding otoferlin, is located on Chromosome 2p23.1 [11]. *OTOF* encodes four transcript isoforms as different translation start sites combined with alternative splicing of some exons. Long isoforms with six C2 domains can be detected in humans and mice, whereas the short isoforms with three C2 domains are only detected in humans [16, 17]. Otoferlin is highly expressed in the inner ear hair cells (IHCs) and vestibule. Otoferlin functions for hearing has been well-characterized. To trigger exocytosis of neurotransmitter in a calcium-dependent manner, Otoferlin interacts with two members of presynaptic SNARE complex (Syntaxin1 and SNAP25) at ribbon synapses of cochlear IHCs, which are incorporated into the membranes of transport vesicles during budding and associated with nerve terminal membranes [18, 19]. These findings revealed that Otoferlin is essential for the synaptic vesicle exocytosis and may acts as a sensor that triggers membrane fusion at the IHC ribbon synapse.

Mutations in the *OTOF* gene have been reported as the main cause of non-syndromic recessive ANSD. To date, more than 100 pathogenic mutations related to subjects with NSHL, including ANSD, have been found in different populations [20]. The c.2485 C > T (p.Q829X) mutation was frequently found in Spanish populations and has also been observed in French, Mexicans, Argentinians and English populations [21–24]. Varga et. al. observed 4 *OTOF* mutations in three non-syndromic recessive auditory neuropathy (NSRAN) families; two nonsense mutations, c.6141G > A and c.6285C > G; and two splice site mutations, IVS39 + 1G > C and 1778delG [25]. There were eleven probable pathogenic variants were found among 160 unrelated ANSD patients in Japan [26]. In China, more than 40 mutations of the *OTOF* gene have been identified. Zhang’s team found 15 novel mutations in 14 congenital ANSD patients [27].

In this study, for the first time, two mutations in the *OTOF* gene were observed in a Chinese family with ANSD by whole-exome sequencing. The otoferlin isoform a is encoded by *OTOF* and consists of 1997 amino acids (aa), including six C2 domains, C2A (2–97 aa), C2B (255–353 aa), C2C (418–529 aa), C2D (961–1068 aa), C2E (1493–1592 aa), and C2F (1733–1863 aa) (Fig. 3a). The c.1273C > T mutation, results in a premature stop codon from arginine to a terminator (p.R425X). It may lead to abnormal or nonfunctional protein products, suggesting it is responsible for the pathogenicity of deafness. Previously, this mutation was reported in a deaf population in Pakistan [28], and this is the first time the mutation was identified in the Chinese population. The novel missense mutation c.4994 T > C, located between the C2E and C2F domains, results in a single amino acid substitution, leucine to proline (p.L1665P). Three pathogenic mutations (E1661K, R1676C, and E1700Q) between C2E and C2F domains have been reported. The missense mutation c.4994 T > C (p.L1665P) was assumed to be pathogenic because 1) mutations in *OTOF* have been reported to be the main cause of autosomal recessive non-syndromic deafness DFNB9 that associated with ANSD [11, 17]; 2) the region is highly conserved across mammalian species (Fig. 3b); 3) it was found in compound heterozygosity with p.R452X; 4) some pathogenic mutations have been found near the mutation site; and 5) no other recessive variants related to deafness could be identified by WES.

In this study, we found two mutations of the *OTOF* gene in a Chinese family with ANSD located in the Guangxi Zhuang Autonomous Region. The findings indicate that there may be rare mutations or genes associated with NSHL in the Zhuang Autonomous Region, which is one of the ethnic minority regions in China. The mutation frequency and hot spots of the deafness gene may differ among disparate ethnic regions and peoples. The findings provide new evidence for investigations of the mutation characteristics of the *OTOF* gene in NSHL in the Zhuang population in China.

## Conclusion

We identified two disease-causing mutations in the *OTOF* gene in a Chinese family with ANSD by WES. Two compound heterozygous mutations of the *OTOF* gene were detected in two affected siblings. Out data indicated that WES is an effective approach for identifying the genetic characteristics of non-syndromic ANSD. The identification of these two mutations extends the mutation spectrum of the *OTOF* gene in the Chinese deaf population. The findings will hopefully facilitate genetic diagnosis and enable otolaryngologists to select appropriate clinical interventions for patients with ANSD.

## Additional file

Additional file 1: Figure S1. Magnetic resonance imaging (MRI) of the inner ear in subject II:3. A single layer scanning of inner ear (left panel) and multilayer 3D reconstruction image (right panel) are shown. (TIF 285 kb)

## Abbreviations

ABR: Brainstem response
AD: Auditory dys-synchrony
AN: Auditory neuropathy
ANSD: Auditory neuropathy spectrum disorder
ASSR: Auditory steady-state response
CM: Cochlear microphonic
DPOAE: Distortion product otoacoustic emission
MRI: Magnetic resonance imaging
NSHL: Non-syndromic hearing loss
WES: Whole-exome sequencing
