# Supplementary figures and images for: Novel compound heterozygous mutations in the OTOF Gene identified by whole-exome sequencing in auditory neuropathy spectrum disorder
Source: BMC Med Genet. 2017 Mar 23;18:35. doi: 10.1186/s12881-017-0400-0 (PMC5364697; doi:10.1186/s12881-017-0400-0)

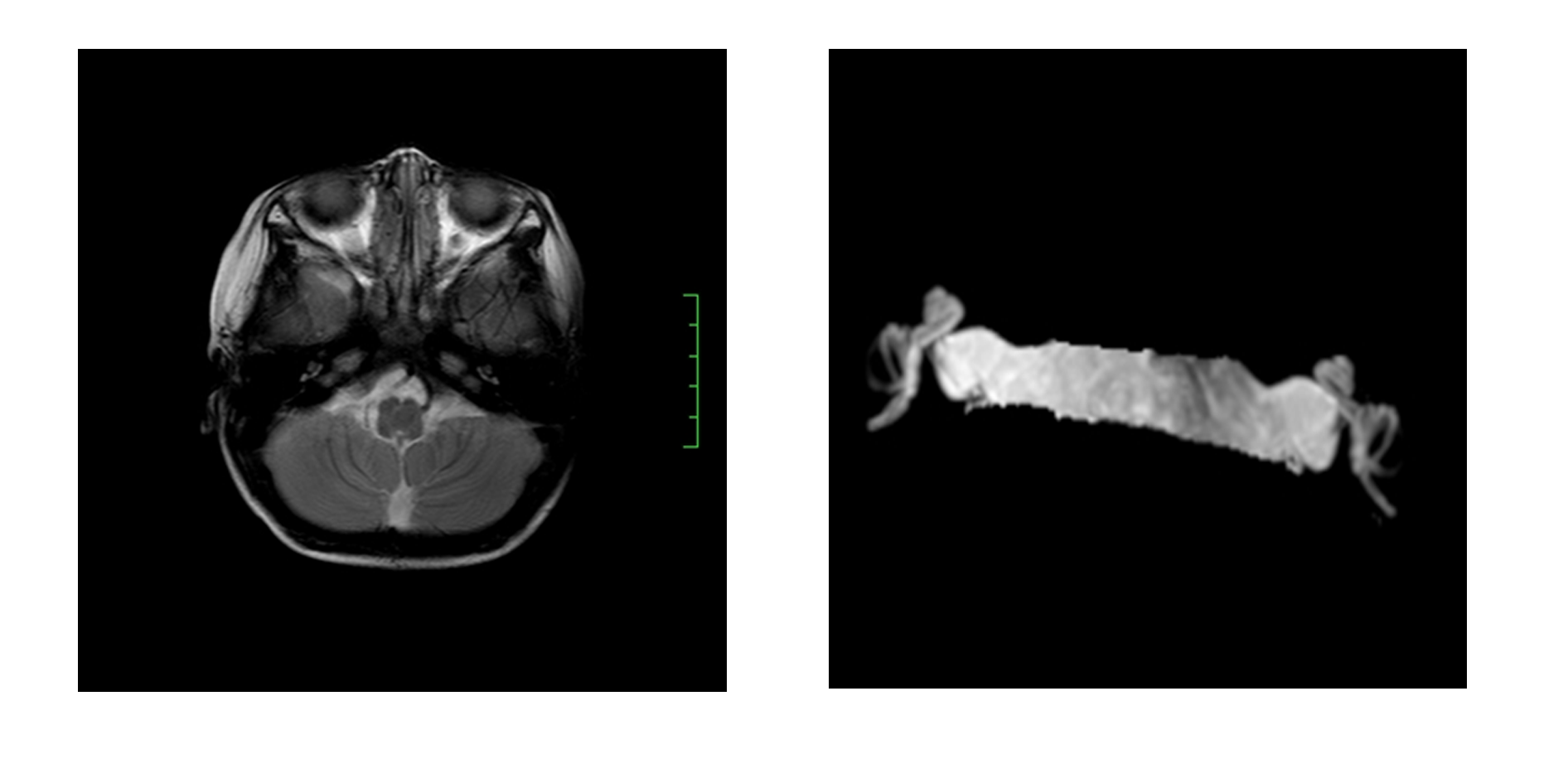

Supplement: Additional file 1: Figure S1. — Magnetic resonance imaging (MRI) of the inner ear in subject II:3. A single layer scanning of inner ear (left panel) and multilayer 3D reconstruction image (right panel) are shown. (TIF 285 kb) [file 12881_2017_400_MOESM1_ESM.tif]
